# Increasing generations in captivity is associated with increased vulnerability of Tasmanian devils to vehicle strike following release to the wild

**DOI:** 10.1038/s41598-017-02273-3

**Published:** 2017-05-19

**Authors:** Catherine E. Grueber, Elizabeth E. Reid-Wainscoat, Samantha Fox, Katherine Belov, Debra M. Shier, Carolyn J. Hogg, David Pemberton

**Affiliations:** 1 School of Life and Environmental Sciences, Faculty of Science, The University of Sydney, Camperdown, NSW Australia; 2 Division of Applied Animal Ecology, San Diego Zoo Institute for Conservation Research, San Diego, CA USA; 3 Save the Tasmanian Devil Program, DPIPWE, Hobart, Tasmania Australia; 4 Department of Ecology & Evolutionary Biology, University of California Los Angeles, Los Angeles, USA; 5 Zoo and Aquarium Association Australasia, Mosman, Australia

## Abstract

Captive breeding of threatened species, for release to the wild, is critical for conservation. This strategy, however, risks producing captive-raised animals with traits poorly suited to the wild. We describe the first study to characterise accumulated consequences of long-term captive breeding on behaviour, by following the release of Tasmanian devils to the wild. We test the impact of prolonged captive breeding on the probability that captive-raised animals are fatally struck by vehicles. Multiple generations of captive breeding increased the probability that individuals were fatally struck, a pattern that could not be explained by other confounding factors (e.g. age or release site). Our results imply that long-term captive breeding programs may produce animals that are naïve to the risks of the post-release environment. Our analyses have already induced changes in management policy of this endangered species, and serve as model of productive synergy between ecological monitoring and conservation strategy.

## Introduction

An important conservation tool for the restoration of threatened populations is captive breeding for reintroduction to the wild^1^. However, adaptation to captivity, via genetically-determined, epigenetic (developmental), or environmental changes, can lead to an individual phenotype that is well-suited to the captive environment, but maladaptive upon release to the wild^2, 3^. Captivity is thought to select against temperaments that may be favoured in the wild, potentially eroding a variety of wild-type behaviors that are essential for survival following release (an overview of studies in this area is provided at Supplementary Table S1). Adaptation to captivity underpinned by genetic changes can occur in just a few years^2^, and may negatively impact conservation management outcomes even before animals are released, if genetic diversity is lost or inbreeding depression goes undetected^4^.

Captive breeding and reintroduction programs have been used worldwide to supplement and/or create new wild populations, with varying degrees of success^5^. Management of captive breeding programs is a delicate balance between multiple goals: preserving genetic diversity^6^, minimising adaptation to captivity^2^, and maintenance of wild behaviors^7^. Although research has shown that wild-caught animals typically fare better upon release than captive-bred animals^8^, sourcing founders from the wild may be impossible if no extant populations remains, or imprudent if removal of sufficient numbers would imperil the source population. Thus, many conservation programs rely on releasing captive-bred animals for species recovery^1^.

Reintroduced animals face a myriad of threats upon release to the wild. Predators, competitors, parasites and inclement weather are all a part of living in an intact ecosystem; anthropogenic threats, such as roads, make life in the wild even more complex. Challenges are exacerbated for captive-bred individuals, as they are raised in closed systems where many of the aforementioned factors have been controlled, often for generations. Loss of behavioral integrity (Supplementary Table S1), and naïveté of innate biological systems to wild environments, can reduce the survival rate of released individuals. Previous studies have reported post-translocation mortality being highest immediately following release, as animals make settlement decisions and identify suitable habitat^9^. Among proposed explanations for high mortality during establishment, “post-release dispersal”, is thought to be an important factor^9^. This mobility increases the likelihood of fatal encounters with predators or anthropogenic threatening processes such as vehicles.

**Figure 1:**
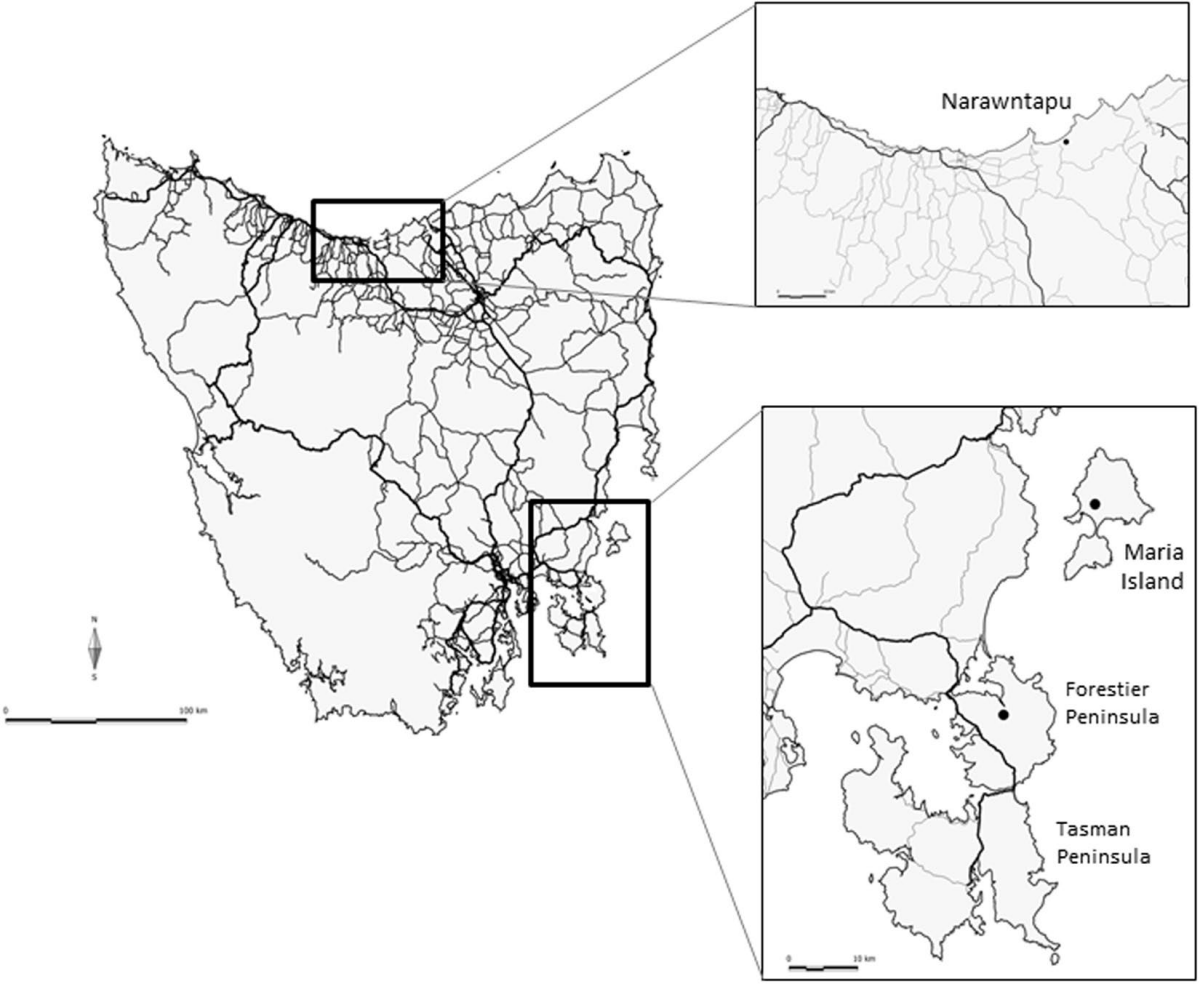
Map of release sites (dots), with roads as marked (highways thick lines, secondary roads thin lines). Figure generated using Manifold System v 8 enterprise edition (www.manifold.net).

The Tasmanian devil (*Sarcophilus harrisii*) is a largely nocturnal carnivorous marsupial endemic to the island state of Tasmania, Australia^10^. The species was considered stable two decades ago, but is now listed as Endangered, due to its rapid decline over the past 20 years as a result of a lethal contagious cancer, devil facial tumour disease (DTFD)^11^. DFTD was first reported in 1996 in NE Tasmania (Fig. 1) and has since spread across the majority of the devil’s native range (DPIPWE, unpubl. data). There has been a >80% decline in devil sightings across Tasmania; with some subpopulations declining by 90% (ref. 11; STDP unpubl. data). To protect against extinction, an insurance population was created in 2006, aimed at establishing a DFTD-free captive population to maintain 95% wild-sourced gene diversity for 50 years^12^. Disease prevalence across the species’ range impacts the devil’s ecological effectiveness as a top-order predator^13^, and increases their susceptibility to stochastic and genetic risks of small population size.

Reintroduction of captive carnivores typically has a high failure rate, with humans being the direct cause of failure in over 50% of documented cases^8^. Nevertheless, for devils, it was determined that supplementation via captive breeding and reintroduction was the only way to prevent inbreeding depression in small isolated populations, and increase numbers in the wild to maintain ecosystem function (STDP unpubl. data). The first releases of captive devils to the wild, in 2012 and 2013 to Maria Island (9,650 ha, Fig. 1), saw unexpectedly high founder survival for a mammalian carnivore^14, 15^. However, Maria Island is a national park with very few anthropogenic threats to the Tasmanian devil. Subsequent to the Maria Island release, there have been three release events at two locations on mainland Tasmania, which are the focus of this analysis (Fig. 1). In contrast to Maria Island, these more recent releases were in human-populated areas with associated infrastructure, including roads (Fig. 1). Devils are vulnerable to vehicles due to their propensity to travel long distances, use roads for dispersal, travel at night and scavenge on roadkill^10^.

The Tasmanian devil insurance metapopulation commenced in 2006 and consists of more than 650 devils held in 35 Australian based zoos, fenced enclosures, an island site and a fenced peninsula. Breeding and transfer recommendations for this metapopulation are made on an annual basis and require a strong rule set to be applied to animal movements due to biosecurity restrictions^12, 16^. Due to the complexity and length of the devil program, our dataset presents a unique opportunity to assess the impact of captive history on individual survival following release to a wild environment as the program has been intensively managed for longer than ten years with detailed record keeping. We test whether individual characteristics, especially generations in captivity, but also age and sex, predict the probability that released animals are struck and killed by vehicles. The results are used to inform current policy, and make management recommendations for reducing road impacts in future releases of devils or other wide-ranging carnivores.

## Methods

All methods were performed in accordance with the relevant guidelines and regulations. All animal work, including releases and monitoring, was undertaken under permit by Save the Tasmanian Devil Program staff under Standard Operating Procedures signed off by the General Manager and the Animal Ethics Committee, and Scientific permit to work with Native Fauna and Flora. Full details of animal and site selections, and post-release monitoring, are provided in Supplementary Methods. In summary, a total of 69 devils were released to two sites: Narawntapu National Park (N = 20 on 25 September 2015) and Forestier Peninsula (N = 39 on 18 November 2015 and N = 10 on 25 February 2016) (Fig. 1). Post-release monitoring variously comprised of trapping, remote sensing cameras and microchip scanners setup at bait stations. Road strike reports were obtained via telephone calls to a state-wide devil “hotline”, and animals collected to confirm ID. For our analysis, we classified all released animals as “known road strike”, “known survivor” (recorded by trapping data up until the 4th of June, 2016, the latest data at the time of analysis) and “unknown” (not recorded beyond 1 day following release). A total of N = 50 released devils had known fates at the end of the study period.

To determine the effects of individual characteristics on vehicle strike vulnerability we extracted relevant data, including sex (S) and age at release (A), from the Tasmanian devil studbook^17^. We used PMx^18^ to evaluate number of generations in captivity (G) for each animal, calculated as the average G of an individual’s parents +1, where population founders are assigned G = 0. Note that G values are not necessarily whole numbers. The Tasmanian devil insurance population operates across a number of institution types, including intensive zoo-based facilities containing one or two individuals per enclosure; managed environmental enclosures (MEEs; group housing with 7 to 10 individuals in less than 5 ha enclosure); and free-range enclosures (FREs; group housing with 10 to 20 individuals in 10 to 22 ha)^12, 16^. We therefore considered whether the type of facility an animal was born in might impact the probability of fatal vehicle strike, although found this parameter to be highly correlated with G (Supplementary Figure S1). In addition, individuals within the insurance population are frequently moved between sites for breeding and operational reasons (Supplementary Table S2). We therefore used G as our measure of variation in captive heritage among individuals.

We identified those individual characteristics that contribute most to probability of road kill using logistic regression. In addition to G, our parameter of interest, the global model included S, A and release site (categorical with two levels; Forestier was the reference category). The response was a binomial 1/0 whether an animal was reportedly killed on a road (=1) or known to have survived (=0). The global model was fitted using the GLM function of R v 3.2.1^19^. Standardisation of predictors and model selection under information theory followed Grueber *et al*.^20^; full details provided in Supplementary Methods.

## Results

**Figure 2:**
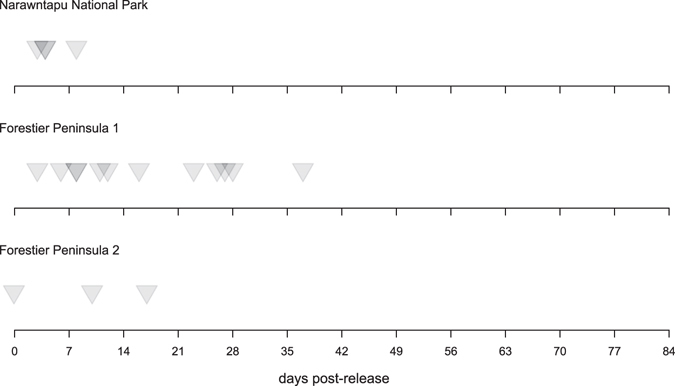
Timeline of devil road strike reports in the 12 weeks of intensive monitoring following each release (Narawntapu National Park release on 25^th^ September 2015; Forestier Peninsula 1 release on 18^th^ November 2015; Forestier Peninsula 2 release on 25^th^ February 2016). Darker intensity of shading for arrows indicates multiple overlaid points.

Our final dataset included 50 animals whose fates were known at the end of the study period, of which 19 were recorded as killed by vehicles on roads, all of which occurred within the first six weeks (Fig. 2). The final dataset included 29 males and 21 females, with an average age of 2.20 (±1.29 SD) years. Of the animals included in our statistical analysis, 36 were released at Forestier Peninsula and 14 released at Narawntapu National Park.

**Figure 3:**
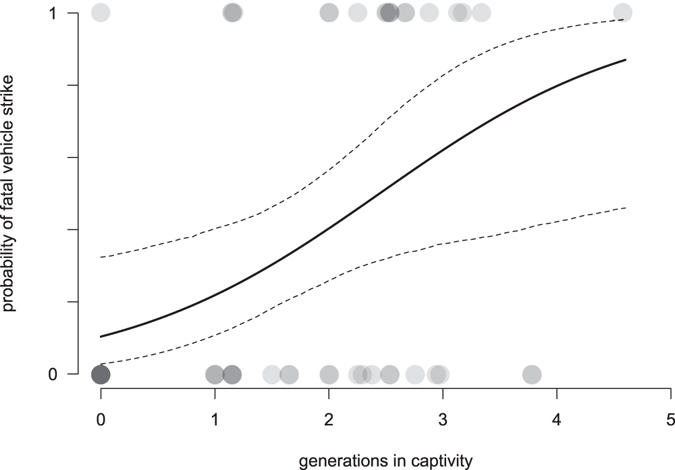
Relationship between generations in captivity and probability that captive Tasmanian devils are struck by vehicles on roads following release. Points indicate observed data, where darker shading indicates multiple overlaid points. The solid line is the fitted relationship, conditional on the mean of other parameters in the model (Table 1); dashed lines are the 95% confidence interval evaluated by parametric bootstrapping.

After model averaging, we found that the only reliable predictor of fatal vehicle strike probability was G (generations in captivity), with increased G dramatically increasing the probability of road strike (Table 1, Fig. 3). Our standardised model allowed us to infer that G has a much stronger effect than sex, age and release site (Table 1). Probability of vehicle strike did not differ between the two release sites as this parameter was poorly supported in the final model (Table 1). Similarly, although age and sex also appeared in the final model, these were poor predictors of vehicle strike probability, as indicated by the large standard errors relative to effect sizes, and low RI values (Table 1).

**Table 1:** Final model (after model averaging; top model set provided at Supplementary Table S3) standardised effects of each predictor on the probability of fatal vehicle strike (*N* = 50).

Predictor^*^	Effect size	Adjusted SE	95% CI	RI
β_0_	−0.581	0.334	−1.236; 0.074	
G	2.108	0.879	0.385; 3.831	1.00
A	1.145	0.864	−0.549; 2.839	0.41
S	0.687	0.694	−0.674; 2.047	0.30
Site(NNP)	0.635	0.901	−1.131; 2.4	0.13

^*^β_0_ = model intercept; A = age; S = sex; G = generations in captivity; Site = release site, FP was the reference category.

It is plausible that the effect of G may be driven largely by differences between wild-born (captive-raised, G = 0) versus captive-born (G > 0) individuals, i.e. that further increase beyond G = 1 has little effect on survival. To test this hypothesis, we excluded all individuals with G = 0 and repeated our analysis with only G > 0 individuals (N = 40). Compared to results that include all animals, G remained the most important parameter in the final model, with an effect in the same direction (although weaker) as the main analysis (Supplementary Table S5). This observation implies that the majority of the negative impacts of captivity are imposed in the first generation, although minor harmful effects may still accumulate.

A total of 19 devils were excluded from our analysis as their fates were not reliably known by the end of the study period (although one devil was non-fatally struck by a car). These devils were proportionally spread across the two sites and of similar mean age (2.21 ± 1.23 SD) and sex ratio (9:10 M:F) to our main dataset. To determine the robustness of our results to these uncertain outcomes, we ran alternative models assuming that all 19 “unknown” animals were either fatally struck by cars, or they all survived. In both cases, G remained a strong predictor of fatal vehicle strike (Supplementary Table S4).

## Discussion

Growing evidence suggests that behavioral and genetic changes mediated by a captive-rearing environment may negatively impact the suitability of captive animals for release. Our results show that these effects can accumulate with increasing generational time in captivity, as evidenced by an increase in vehicle strike susceptibility with increasing pedigree depth in captive-released Tasmanian devils. This result has wide-ranging implications for decision-making around devil releases into the future, as well as release planning for other captive bred species. Our results support recommendations that releases from captive programs be conducted as soon after founding as operationally possible, so that generations in captivity are minimised^2^.

Differences in behavioral expression at the individual level can result from combined influence of genetics, epigenetics and environmental effects^21^, so it is crucial to investigate the mechanisms underpinning such adaptation to captivity. Although we have identified a strong association between generations in captivity and susceptibility to vehicle strike upon release, we were unable to determine whether this pattern is the result of genetic changes in captivity, or accumulated non-genetic changes in behavior. Broad genetic changes have the potential to negatively impact recipient populations^22^, and such risks must be weighed against risks from the alternatives, including not supplementing fragmented and dwindling populations. Individual behavioural changes are not surprising given that animals in captivity are often protected from disease, predation, starvation, competition and environmental extremes. It is possible that a lack of cognitive stimulation in captivity may produce captive-reared individuals that exhibit difficulty learning or adapting to novel experiences^23^, although an earlier release of devils onto Maria Island, with fewer anthropogenic threats, was successful, with devils adapting quickly to that site^14, 15^. Nevertheless, these skills are critical for vehicle avoidance behavior, which includes object detection, threat assessment and evasion, and may be negatively impacted by a lifetime of habituation towards humans and vehicles, as a result of daily exposure in captive facilities, allowing for a neutral or even positive association to vehicles, or a lack of negative experiences with vehicles prior to release. Thus, to improve devil release outcomes, individuals should be selected for release that have had minimal time in captivity and/or behavioral training could be utilized to provide the experiences necessary for captive devils to develop a negative association with vehicles.

Roads are a major threatening process for this species^24^, with annual roadkill rates documented at one site between 2010 and 2013 of 8.8 ± 2.1% (STDP unpublished data). Comparatively, the rate of devil roadkill we observed following translocation was high. Although it was predicted that some fatalities from vehicle strikes may occur as a result of the generally high rate of roadkill seen across Tasmania^24^, the high percentage during the first few weeks following release exceeded expectations. The clustering of roadkill events during the first few weeks post-release (Fig. 2), suggests that this early period represents a learning phase for devils, while they acclimate to the anthropogenic landscape. While sex and age did not impact survivorship, it is unclear whether these early road strikes occur at random in regards to other traits, for example some individuals may be disproportionately susceptible relative to others, who may learn to avoid vehicles. Nevertheless, road strike for the released animals was very low after the initial 6-week post-release period, suggesting that, when considered as a group, devils increasingly avoided roads.

Such high rates of road strike following release indicate a vital need to expedite research around various factors that influence susceptibility to this threat post-release, to improve policy decisions around the use of captive-bred animals for supplementing wild sites, and maximise survival during future translocations. The aim of the STDP is to translocate across the existing range of the devil, the vast majority of which is within 20 km (devil dispersal distance) of a road. Thus, site selection to avoid roads altogether is not possible, and ongoing work to identify the mechanisms that underpin variation among individual susceptibility to roads is invaluable for making release selections in the future.

In addition to the current study, current research is targeting the impacts of captivity on devil phenotypic diversity to provide quantitative guidelines for future releases. Results from these studies could aid in the development of husbandry protocols that minimize animal exposure to humans and vehicles, especially in association to food. In addition, development of enrichment strategies could help inculcate natural behaviour^25^ and aversive stimulus training could be utilized as a pre-release strategy to ensure the release of behaviourally competent devils with a strong negative association with vehicles^26^. However, the most immediate programmatic adaptations in response to this study’s findings include policy changes in regards to release management. In particular, future translocations will use pedigree statistics to rank animals when selecting a release cohort. Staged releases via more natural sites, such as Maria Island, may assist in preventing habituation/attraction to vehicles prior to release to the Tasmanian mainland and release strategies such as familiarization^27, 28^ and conspecific cueing^9, 28^ could reduce dispersal and increase survival post-release (see Supplementary Methods). Collectively, these efforts will facilitate the recovery of the devil in its native range, as well as provide guidance for other captive-release programs, particularly for wide-ranging carnivores.

We have observed a strong effect of captive breeding on the probability that devils will be fatally struck by cars in the period immediately following their release to wild environments. Like devils, wild release is a key goal of many conservation programs, and threats such as vehicles cannot always be fully mitigated. Our results offer insights to improve animal survival following release, especially for wide-ranging species. In particular, conservation breeding programs should strive to have as short a captive period as possible, when those populations are to be used as a source for releases. We also recommend utilising individual pedigree statistics, such as generations in captivity, to inform selection of individuals for release where lower values may represent animals with greater propensity to survive following release. Further research exploring novel strategies for release planning, and the genetic and non-genetic mechanisms underpinning changes over time in captivity will help to better understand the underlying processes that have driven these patterns in devils, and offer solutions to improving the success of captive releases of devils and other species.

## Electronic supplementary material

Supplementary Material
