# Supplementary material for: Increasing generations in captivity is associated with increased vulnerability of Tasmanian devils to vehicle strike following release to the wild
Source: Sci Rep. 2017 May 19;7:2161. doi: 10.1038/s41598-017-02273-3 (PMC5438407; doi:10.1038/s41598-017-02273-3)
Supplement: Supplementary file 1 — Supplementary Material [file 41598_2017_2273_MOESM1_ESM.doc]

**Increasing generations in captivity is associated with increased vulnerability of Tasmanian devils to vehicle strike following release to the wild.**

Catherine E. Grueber, Elizabeth E. Reid-Wainscoat, Samantha Fox,
Katherine Belov, Debra M. Shier, Carolyn J. Hogg, David Pemberton

## Supplementary Methods

#### Release site selection and translocations

Because multiple biological and anthropogenic factors may influence post-release survival of devils, the STDP implemented a number of roadkill mitigation measures including remote site location, soft release tactics (feed stations), dampening dispersal trials (familiarization [following Shier 2006] or conspecific cueing [following Stamps 1988]), employment of virtual fence technology, signage on major roads, and public outreach. The results of these activities are to be reported elsewhere.

Narawntapu National Park: The first release of captive devils into a Tasmanian mainland site was conducted on the 25^th^ of September, 2015, at Narawntapu National Park in the north of the State (Figure 1). Narawntapu was chosen as a release site due to its abundant devil population prior to DFTD arriving in 2008, with the site thereby inferred to provide suitable habitat. Following DFTD arrival, the wild incumbent population has reduced to approximately 15-20 animals (SF unpubl. data) with genetic diversity beginning to decline (CEG unpubl. data). Additionally, being a National Park, the risk of some threats such as roadkill, dogs and persecution are reduced relative to other sites in Tasmania.

Forestier Peninsula: The second and third releases occurred onto the Forestier Peninsula in the south-east of the State on the 18^th^ of November, 2015, and the 25^th^ of February, 2016, respectively. Forestier Peninsula has been a long-term study site for the STDP including monitoring DFTD spread and the effect of the disease on the incumbent population (e.g. Ujvari et al. 2014). In 2012, all devils were removed from Forestier Peninsula; two years of follow-up monitoring ensured that all devils had been removed. A barrier was erected at the isthmus between the town of Dunalley and the Forestier Peninsula to prevent the movement of diseased devils onto the now disease-free Forestier Peninsula. The Tasman peninsula, at the southern tip of Forestier Peninsula (Figure 1), still has a DFTD-free incumbent devil population. There appears to be limited movement between Forestier and Tasman Peninsula populations based on genetic analysis and trapping surveys (STDP unpubl. data).

#### Animal selection for releases

Devils were selected for release based on their age, sex, birth location, mean kinship relative to the remaining individuals in the insurance population (based on the Tasmanian devil studbook [Srb 2015]) and minimal mean kinship to other devils in their release group. Tasmanian devils in the insurance population are housed in three types of enclosure: intensive – zoo-based facilities containing one or two individuals; managed environmental enclosures (MEEs) – group housing with 7 to 10 individuals in less than 5 ha; and free-range enclosures (FREs) – group housing with 10 to 20 individuals in 10 to 22 ha (Hogg and Lee 2014; Hogg et al. 2016).

Narawntapu National Park: One of the STDP’s goals for the first release of Tasmanian devils to mainland Tasmania was to assess the effectiveness of a DFTD immunisation protocol (Woods et al. 2007); all devils selected for this release were immunised as part of ongoing work for a different study. Two different cohorts were selected – individuals that had been captive-raised, i.e. they had lived in a DFTD environment until at least one year of age (N = 11), or came into the insurance population as pouch young (N = 1); the other cohort was captive-born, i.e. individuals from over-represented lines in the insurance population (Hogg et al. 2015) (N = 8). The Narawntapu release group totalled 20 individuals with a male:female sex ratio of 11:9, and an age structure of age 1 (N = 5); age 2 (N = 2); age 3 (N = 2); and age 4 (N = 11). The age 4 animals were all captive-raised, having been born in the wild (N = 12); while the younger cohort (ages 1 to 3) were captive-born in intensive facilities (N = 2) or FREs (N = 7).

Forestier Peninsula: Each of the two releases to Forestier Peninsula consisted of captive-born devils (except for one wild-born, captive-raised animal). The first release consisted of 39 individuals from intensive (N = 10), MEEs (N = 23) and FREs (N = 6); with a sex ratio of 27:22 males:females and an age structure of age 1 (N = 12); age 2 (N = 15); age 3 (N = 8); and age 4 (N = 4). The second release comprised dispersing-age juvenile devils of sex ratio7:3 males:females; males were born in FREs and females in an intensive facility.

#### Post-release monitoring

All released animals were uniquely microchipped with ISO-standard companion animal injectable glass transponders, and had a unique bleach mark painted on their rump to enable visual identification of each devil on camera. Post-release monitoring involved the use of trapping, remote sensing cameras and microchip scanners setup at bait stations. Bait stations involved wallaby, possum or kangaroo carcasses being staked to the ground, and a camera set up to record visitations by devils. Narawntapu National Park had five bait stations set up in an east – west direction across the park, which were rebaited every week. Forestier Peninsula had eight bait stations that were rebaited weekly. Bait stations and the resultant camera and microchip scanners, remained in place for 12 weeks. Trapping trips took place over seven days using specially made devil traps made from polypipe tubing usually used for drainage (Mooney and Ralph, unpublished design). Forty traps were set out across the release site, with traps baited with lamb flaps or wallaby pieces. Traps were checked daily, and four trapping trips occurred over the 12 week post-release period. Each devil caught was given a full body health check including taking weight and recording any wounds. We used these three forms of monitoring (cameras, microchip scanners and trapping), to construct movement patterns of each devil following release. In addition, the Save the Tasmanian Devil Program has a local “hotline”, which has a 24 hour response, to take public reports of roadkill and welfare cases of animals with advanced DFTD (state-wide).

For our analysis we classified all released animals as “known road strike”, “known survivor” and “unknown”. Animals notified through the Save the Tasmanian Devil Program hotline were collected to enable confirmation of individual ID. All roadkill reports occurred within six weeks post-release, with the exception of one Forestier release animal killed by a vehicle after 14 weeks, and one Narawntapu release animal killed after nine months. The long period prior to roadkill reports for the latter two animals suggests that these events are unlikely to have been associated with initial exploratory behavior, therefore these two animals were considered to have “survived” the initial establishment period. Including these animals as “road strike” did not qualitatively change our results (data not shown). Other animals were designated as “known survivor” if they were observed via trapping, camera and microchip data (from both sites) up until the 4^th^ of June, 2016 (most recent data at time of writing). Animals that were not recorded on any data type, beyond 1 day following release, were recorded as “unknown” and excluded from the analysis.

#### Model selection

To facilitate inference across predictors on different measurement scales, model predictors were standardised by subtracting the mean and dividing by 2 SD (following Gelman 2008). Model selection was conducted under an information theoretic approach, taking all submodels of the global model and averaging the top 2 AIC_C_ (corrected Akaike information criterion) by the conditional average method using the R-package MuMIn (Bartoń 2009). We report the final model effect sizes and their 95% confidence intervals (based on 1.96 × adjusted SE), as well as the relative importance of each parameter (RI): the posterior probability that a parameter is included in the best model, evaluated by summing the Akaike weights of all submodels containing that parameter. To facilitate interpretation, model fitted values were backtransformed onto the natural scale and 95% confidence intervals generated by parametric bootstrapping.

#### Literature cited in Supplementary Text

## Supplementary Material


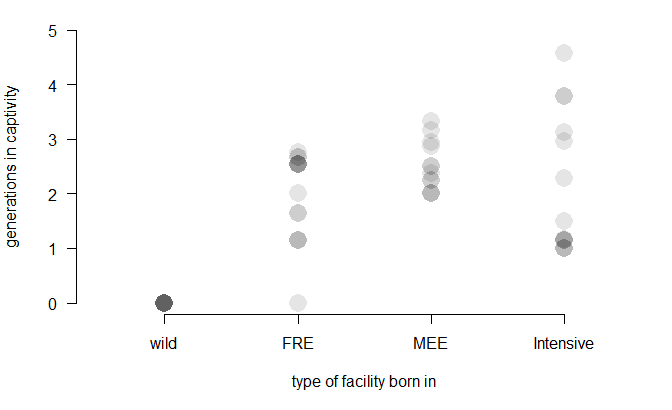


**Supplementary Figure S1** Relationship between the type of facility an animal is born in, and its generational depth (*N* = 50). Four sites of origin are shown: wild, FRE (free-range enclosure; group housing with 10 to 20 individuals in 10 to 22 ha), MEE (managed environmental enclosure; group housing with 7 to 10 individuals in less than 5 ha) and intensive (zoo-based facilities containing one or two individuals), which together represent a continuum of increasing intensiveness and decreasing enclosure size (increasing housing density) (see also [Hogg et al. 2015](#_ENREF_19)).

**Supplementary Table S1** Evidence of behavior changes in response to captivity. Comments in parentheses indicate data collected over multiple generations.

|  | | Behavior affected | | | | | | | | | | | | | | | |  | |  |
| --- | --- | --- | --- | --- | --- | --- | --- | --- | --- | --- | --- | --- | --- | --- | --- | --- | --- | --- | --- | --- |
| Species | | Antipredator | | Foraging | | Competitive ability; aggression | | Locomotion; activity | | Spatial orientation; refuge use | | Maternal care | | Mate choice | | Social behavior | | References | |  |
| Environmental effects of captive environment on behavior (captive versus wild) | | | | | | | | | | | | | | | | | | | |  |
|  | Rainbow darter (*Etheostoma caeruleum*) | | * | | * | |  | |  | |  | |  | |  | |  | | Crane, Lampe & Mathis, 2015 |  |
|  | Brown trout (*Salmo trutta*) | |  | | * | | * | |  | |  | |  | |  | | * | | Sundstrom, Bohlin & Johnsson, 2004; Sundstrom & Johnsson, 2001; Sundstrom, Lohmus & Johnsson, 2003 |  |
|  | Coho salmon (*Oncorhynchus*) | |  | |  | | * | |  | |  | |  | |  | |  | | Swain & Riddell, 1990 |  |
|  | Steelhead trout (*Oncorhynchus mykiss*) | | * | |  | | * | | * | |  | |  | |  | |  | | Berejikian, 1995; Berejikian, Mathews & Quinn, 1996; Lee & Berejikian, 2008 |  |
|  | Wild cod (*Gobiusculus flavescens*) | |  | | * | |  | |  | |  | |  | |  | |  | | Steingrund & Ferno, 1997 |  |
|  | Rhea (*Rhea americana*) | | * | |  | |  | |  | |  | |  | |  | |  | | de Azevedo & Young, 2006 |  |
|  | Parrots and Passerines (various species) | | * | |  | |  | |  | |  | |  | |  | |  | | Carrete & Tella, 2015 |  |
|  | Kangaroo rat (*Dipodomys heermanni*) | | * | |  | |  | |  | |  | |  | |  | |  | | Yoerg & Shier, 1997 |  |
|  | Black-tailed prairie dog (*Cynomys ludovicianus*) | | * | |  | |  | |  | |  | |  | |  | |  | | Shier & Owings, 2006; Shier & Owings, 2007 |  |
| Behavior modified in response to generations in captivity | | | | | | | | | | | | | | | | | | | |  |
| *Invertebrates* | | |  | |  | |  | |  | |  | |  | |  | |  | |  |  |
|  | Coonstripe shrimp (*Panadalus danae*) | | *(10) | |  | |  | |  | |  | |  | |  | |  | | Marliave, Gergits & Aota, 1993 |  |
| *Fish* | |  | |  | |  | |  | |  | |  | |  | |  | |  | | |
|  | Crimson spotted rainbowfish (*Melanotaenia duboulayi*) |  | |  | |  | |  | |  | |  | |  | | * (many) | | Kydd & Brown, 2009 | | |
|  | Atlantic salmon (*Salmo salar*) |  | | *(many) | |  | |  | |  | |  | |  | |  | | Thodesen *et al.*, 1999 | | |
|  | Masu salmon (*Oncorhynchus masou*) | *(30) | |  | |  | |  | |  | |  | |  | |  | | Yamamoto & Reinhardt, 2003 | | |
|  | Brown trout (*Samo trutta*) |  | | *(many) | |  | |  | |  | |  | |  | |  | | Sanchez *et al.*, 2001 | | |
|  | *Herps* |  | |  | |  | |  | |  | |  | |  | |  | |  | | |
|  | Mallorcan midwife toad (*Alytes muletensis*) | *(9-12) | |  | |  | |  | |  | |  | |  | |  | | Kraaijeveld-Smit *et al.*, 2006 | | |
|  | Otago skink (*Oligosoma otagense*) |  | |  | |  | | *(1-3) | |  | |  | |  | |  | | Connolly & Cree, 2008 | | |
| *Birds* | |  | |  | |  | |  | |  | |  | |  | |  | |  | | |
|  | Red junglefowl (*Gallus gallus*) | * (4) | |  | |  | |  | |  | |  | |  | |  | | Campler, Jongren & Jensen, 2009; Hakansson & Jensen, 2008 | | |
| *Mammals* | |  | |  | |  | |  | |  | |  | |  | |  | |  | | |
|  | Golden lion tamarin (*Leontopithecus rosalia*) | * | | * | |  | | * | | * | | * | |  | |  | | Kleiman *et al.*, 1990 | | |
|  | House mouse (*Mus musculus*) |  | |  | |  | |  | |  | |  | | *(3) | |  | | Slade *et al.*, 2014 | | |
|  | Old field mous (*Peromyscus poloniotus*) | *(1-3) | |  | |  | | *(1-3) | | *(1-3) | |  | |  | |  | | McPhee, 2003; McPhee, 2004 | | |
|  | Norway rat (*Rattus norvegicus*) |  | |  | |  | |  | |  | |  | |  | |  | |  | | |
| Behavior loss in captivity | | | | | | | | | | | | | | | | | | | | |
|  | Ratsnake (*Elaphe obsoleta*) |  | | * | |  | |  | |  | |  | |  | |  | | DeGregorio *et al.*, 2013 | | |
|  | Kangaroo rat (*Dipodomys heermanni*) | * | |  | |  | |  | |  | |  | |  | |  | | Yoerg & Shier, 1997 | | |
|  | Red-legged partridge (*Alectoris rufa*) | * | |  | |  | |  | |  | |  | |  | |  | | Gaudioso *et al.*, 2011 | | |

#### Literature cited in Supplementary Table S1

Berejikian B.A. (1995) The effects of hatchery and wild ancestry and experience on the relative ability of steelhead trout fry (*Oncorhynchus mykiss*) to avoid a benthic predator. *Canadian Journal of Fisheries and Aquatic Sciences* **52**, 2476-2482.

Berejikian B.A., Mathews S.B., Quinn T.P. (1996) Effects of hatchery and wild ancestry and rearing environments on the development of agonistic behavior in steelhead trout (*Oncorhynchus mykiss*) fry. *Canadian Journal of Fisheries and Aquatic Sciences* **53**, 2004-2014.

Campler M., Jongren M., Jensen P. (2009) Fearfulness in red junglefowl and domesticated White Leghorn chickens. *Behavioural Processes* **81**, 39-43.

Carrete M., Tella J.L. (2015) Rapid loss of antipredatory behaviour in captive-bred birds is linked to current avian invasions. *Scientific Reports* **5**, 18274.

Connolly J.D., Cree A. (2008) Risks of a late start to captive management for conservation: Phenotypic differences between wild and captive individuals of a viviparous endangered skink (*Oligosoma otagense*). *Biological Conservation* **141**, 1283-1292.

Crane A.L., Lampe M.J., Mathis A. (2015) Maladaptive behavioural phenotypes in captive-reared darters (Etheostomacaeruleum, Storer 1845). *Journal of Applied Ichthyology* **31**, 787-792.

de Azevedo C.S., Young R.J. (2006) Shyness and boldness in greater rheas *Rhea americana* Linnaeus (Rheiformes, Rheidae): the effects of antipredator training on the personality of the birds. *Revista Brasileira de Zoologia* **23**, 202-210.

DeGregorio B.A., Weatherhead P.J., Sperry J., Tuberville T. (2013) Time in captivity affects foraging behavior of ratsnakes: implications for translocation. *Herpetological Conservation and Biology* **8**, 581-590.

Gaudioso V.R., Sánchez-García C., Pérez J.A., Rodríguez P.L., Armenteros J.A., Alonso M.E. (2011) Does early antipredator training increase the suitability of captive red-legged partridges (*Alectoris rufa*) for releasing? *Poultry Science* **90**, 1900-1908.

Hakansson J., Jensen P. (2008) A longitudinal study of antipredator behaviour in four successive generations of two populations of captive red junglefowl. *Applied Animal Behaviour Science* **114**, 409-418.

Kleiman D.G., Beck B.B., Baker A., Ballou J.D., Dietz L., Dietz J. (1990) The conservation program for the golden lion tamarin, *Leontopithecus rosalia*. *Endangered species update* **8**, 82-85.

Kraaijeveld-Smit F.J.L., Griffiths R.A., Moore R.D., Beebee T.J.C. (2006) Captive breeding and the fitness of reintroduced species: a test of the responses to predators in a threatened amphibian. *Journal of Applied Ecology* **43**, 360-365.

Kydd E., Brown C. (2009) Loss of shoaling preference for familiar individuals in captive-reared crimson spotted rainbowfish *Melanotaenia duboulayi*. *Journal of Fish Biology* **74**, 2187-2195.

Lee J.S.F., Berejikian B.A. (2008) Effects of the rearing environment on average behaviour and behavioural variation in steelhead. *Journal of Fish Biology* **72**, 1736-1749.

Marliave J.B., Gergits W.F., Aota S. (1993) F10 pandalid shrimp: Sex determination; DNA and dopamine as indicators of domestication; and outcrossing for wild pigment pattern. *Zoo Biology* **12**, 435-451.

McPhee M.E. (2003) Generations in captivity increases behavioral variance: considerations for captive breeding and reintroduction programs. *Biological Conservation* **115**, 71-77.

McPhee M.E. (2004) Morphological change in wild and captive oldfield mice *Peromyscus polionotus subgriseus*. *Journal of Mammalogy* **85**, 1130-1137.

Sanchez M.P., Chevassus B., Labbe L., Quillet E., Mambrini M. (2001) Selection for growth of brown trout (*Salmo trutta*) affects feed intake but not feed efficiency. *Aquatic Living Resources* **14**, 41-48.

Shier D.M., Owings D.H. (2006) Effects of predator training on behavior and post-release survival of captive prairie dogs (*Cynomys ludovicianus*). *Biological Conservation* **132**, 126-135.

Shier D.M., Owings D.H. (2007) Effects of social learning on predator training and post-release survival in juvenile black-tailed prairie dogs (*Cynomys ludovicianus*). *Animal Behaviour* **73**, 567-577.

Slade B., Parrott M.L., Paproth A., Magrath M.J.L., Gillespie G.R., Jessop T.S. (2014) Assortative mating among animals of captive and wild origin following experimental conservation releases. *Biology Letters* **10**, 4.

Steingrund P., Ferno A. (1997) Feeding behaviour of reared and wild cod and the effect of learning: Two strategics of feeding on the two-spotted goby. *Journal of Fish Biology* **51**, 334-348.

Sundstrom L.F., Bohlin T., Johnsson J.I. (2004) Density-dependent growth in hatchery-reared brown trout released into a natural stream. *Journal of Fish Biology* **65**, 1385-1391.

Sundstrom L.F., Johnsson J.I. (2001) Experience and social environment influence the ability of young brown trout to forage on live novel prey. *Animal Behaviour* **61**, 249-255.

Sundstrom L.F., Lohmus M., Johnsson J.I. (2003) Investment in territorial defence depends on rearing environment in brown trout (*Salmo trutta*). *Behavioral Ecology and Sociobiology* **54**, 249-255.

Swain D.P., Riddell B.E. (1990) Variation in agonistic behavior between newly emerged juveniles from hatchery and wild populations of coho salmon, *Oncorhynchus kisutch*. *Canadian Journal of Fisheries and Aquatic Sciences* **47**, 566-571.

Thodesen J., Grisdale-Helland B., Helland S.J., Gjerde B. (1999) Feed intake, growth and feed utilization of offspring from wild and selected Atlantic salmon (*Salmo salar*). *Aquaculture* **180**, 237-246.

Yamamoto T., Reinhardt U.G. (2003) Dominance and predator avoidance in domesticated and wild masu salmon Oncorhynchus masou. *Fisheries Science (Tokyo)* **69**, 88-94.

Yoerg S.I., Shier D.M. (1997) Maternal presence and rearing condition affect responses to live predator in kangaroo rats (*Dipodomys heermanni arenae*). *Journal of Comparative Psychology* **111**, 362-369.

**Supplementary Table S2** Chart indicating the type of facility inhabited, across the lifespan, by devils released to the wild (green = wild, blue = FRE [free-range enclosure; group housing with 10 to 20 individuals in 10 to 22 ha], pink = MEE [managed environmental enclosure; group housing with 7 to 10 individuals in less than 5 ha], orange = intensive [zoo-based facilities containing one or two individuals]). Data are sorted first by road-kill outcome, then by generation (G).

|  |  |  |  | Type of facility inhabited at each age, prior to release | | | | |
| --- | --- | --- | --- | --- | --- | --- | --- | --- |
| ID | Sex | G | Road kill^*^ | Age 0-1 | Age 1-2 | Age 2-3 | Age 3-4 | Age 4-5 |
| 1334 | F | 0.0000 | 0 |  |  |  |  |  |
| 1436 | M | 0.0000 | 0 |  |  |  |  |  |
| 1437 | M | 0.0000 | 0 |  |  |  |  |  |
| 1439 | M | 0.0000 | 0 |  |  |  |  |  |
| 1447 | M | 0.0000 | 0 |  |  |  |  |  |
| 1450 | M | 0.0000 | 0 |  |  |  |  |  |
| 1456 | F | 0.0000 | 0 |  |  |  |  |  |
| 1464 | M | 0.0000 | 0 |  |  |  |  |  |
| 1763 | M | 0.0000 | 0 |  |  |  |  |  |
| 1341 | F | 1.0000 | 0 | ‡ |  | † |  |  |
| 1342 | F | 1.0000 | 0 | ‡ |  | † |  |  |
| 1346 | F | 1.0000 | 0 | ‡ |  |  |  |  |
| 1579 | M | 1.1500 | 0 |  |  |  |  |  |
| 1581 | F | 1.1500 | 0 |  |  |  |  |  |
| 1583 | M | 1.1500 | 0 |  |  |  |  |  |
| 1585 | M | 1.1500 | 0 |  |  |  |  |  |
| 1198 | F | 1.5000 | 0 |  |  | † |  |  |
| 1591 | F | 1.6500 | 0 |  |  |  |  |  |
| 1688 | M | 1.6500 | 0 |  |  |  |  |  |
| 1434 | M | 2.0000 | 0 |  |  |  |  |  |
| 1523 | M | 2.0000 | 0 |  |  |  |  |  |
| 1555 | F | 2.2500 | 0 |  |  |  |  |  |
| 1810 | F | 2.2905 | 0 |  |  |  |  |  |
| 1641 | F | 2.3750 | 0 |  |  |  |  |  |
| 1749 | M | 2.5313 | 0 |  |  |  |  |  |
| 1762 | M | 2.5313 | 0 |  |  |  |  |  |
| 1764 | M | 2.7523 | 0 |  |  |  |  |  |
| 1628 | F | 2.9375 | 0 |  |  |  |  |  |
| 1360 | M | 2.9688 | 0 |  | † |  |  |  |
| 1816 | F | 3.7749 | 0 |  |  |  |  |  |
| 1818 | F | 3.7749 | 0 |  |  |  |  |  |
| 1449 | M | 0.0000 | 1 |  |  |  |  |  |
| 1584 | M | 1.1500 | 1 |  |  |  |  |  |
| 1686 | F | 1.1500 | 1 |  |  |  |  |  |
| 1592 | F | 1.1650 | 1 |  |  |  |  |  |
| 1297 | M | 2.0000 | 1 |  |  |  |  |  |
| 1476 | F | 2.0000 | 1 |  |  |  |  |  |
| 1302 | M | 2.2500 | 1 |  |  |  |  |  |
| 1300 | M | 2.5000 | 1 |  |  |  |  |  |
| 1645 | M | 2.5000 | 1 |  |  |  |  |  |
| 1744 | M | 2.5313 | 1 |  |  |  |  |  |
| 1745 | M | 2.5313 | 1 |  |  |  |  |  |
| 1750 | M | 2.5313 | 1 |  |  |  |  |  |
| 1606 | F | 2.6667 | 1 |  |  |  |  |  |
| 1608 | M | 2.6667 | 1 |  |  |  |  |  |
| 1151 | F | 2.8750 | 1 |  |  |  |  |  |
| 1662 | M | 3.1250 | 1 |  |  |  |  |  |
| 1309 | F | 3.1667 | 1 |  |  |  |  |  |
| 1629 | F | 3.3333 | 1 |  |  |  |  |  |
| 1704 | M | 4.5714 | 1 |  |  |  |  |  |
| 1445 | F | 0.0000 | U |  |  |  |  |  |
| 1463 | F | 0.0000 | U |  |  |  |  |  |
| 1145 | F | 1.0000 | U |  |  |  |  |  |
| 1586 | M | 1.1500 | U |  |  |  |  |  |
| 1537 | M | 1.5000 | U |  | † |  |  |  |
| 1549 | F | 1.5000 | U |  | † |  |  |  |
| 1690 | M | 1.6500 | U |  |  |  |  |  |
| 1138 | M | 2.0000 | U |  |  |  |  |  |
| 1225 | F | 2.0000 | U |  |  |  | † |  |
| 1367 | F | 2.0000 | U |  |  |  |  |  |
| 1526 | M | 2.0000 | U |  |  |  |  |  |
| 1531 | M | 2.0000 | U |  |  |  |  |  |
| 1533 | M | 2.2500 | U |  |  |  |  |  |
| 1660 | M | 2.3750 | U |  |  |  |  |  |
| 1635 | F | 2.5000 | U |  |  |  |  |  |
| 1246 | F | 2.5234 | U |  |  |  |  |  |
| 1631 | F | 2.6250 | U |  |  |  |  |  |
| 1607 | M | 2.6670 | U |  |  |  |  |  |
| 1649 | F | 2.7500 | U |  |  |  |  |  |

^*^ 0 = known to have survived, 1 = known to have been hit by a car, U = unknown fate

^†^ devils were moved to a FRE for the 2 months prior to their release

^‡^ devils brought into the insurance population as pouch young, so although technically born in the wild, they did not become independent until they were already present in the insurance population.

**Supplementary Table S3** Top model set (2 AIC_C_) of generalized linear models for probability of fatal vehicle strike following release of captive Tasmanian devil (*Sarcophilus harrisii*); final model provided at Table 2.

| Model^*^ | AIC_C_ | ∆AIC_C_ | *w_i_* |
| --- | --- | --- | --- |
| β_0_ + G | 63.117 |  | 0.244 |
| β_0_ + A + G | 63.495 | 0.378 | 0.202 |
| β_0_ + S + G | 64.491 | 1.374 | 0.123 |
| β_0_ + A + S + G | 64.574 | 1.457 | 0.118 |
| β_0_ + Site(NNP) + G | 64.851 | 1.735 | 0.103 |

^*^ β_0_ = model intercept; A = age; S = sex; G = generations in captivity; Site = release site, Forestier was the reference category

**Supplementary Table S4** Final model results (after model averaging) under alternative assumptions regarding the 19 “unknown” fate animals excluded from the main analysis. Each model presents standardised effects of each predictor on the probability of fatal vehicle strike (*N* = 69)

| Model | Predictor^*^ | Effect size | Adjusted SE | 95% CI | RI |
| --- | --- | --- | --- | --- | --- |
| Assuming all unknowns died | β_0_ | 0.228 | 0.261 | -0.283; 0.739 |  |
|  | G | 1.541 | 0.679 | 0.211; 2.872 | 1.00 |
|  | A | 0.903 | 0.674 | -0.418; 2.224 | 0.38 |
|  | Site(NNP) | 0.552 | 0.678 | -0.777; 1.881 | 0.20 |
| Assuming all unknowns survived | β_0_ | -1.125 | 0.314 | -1.741; -0.509 |  |
|  | G | 1.903 | 0.776 | 0.382; 3.424 | 1.00 |
|  | A | 0.963 | 0.735 | -0.478; 2.403 | 0.46 |
|  | S | 0.704 | 0.622 | -0.514; 1.923 | 0.40 |

^*^ β_0_ = model intercept; A = age; S = sex; G = generations in captivity; Site = release site, FP was the reference category

**Supplementary Table S5** Final model (after model averaging) standardised effects of each predictor on the probability of fatal vehicle strike, excluding wild-born individuals where G = 0 (*N* = 40).

| Predictor^*^ | Effect size | Adjusted SE | 95% CI | RI |
| --- | --- | --- | --- | --- |
| β_0_ | -0.218 | 0.349 | -0.901; 0.465 |  |
| G | 1.306 | 0.780 | -0.224; 2.836 | 0.74 |
| A | 1.199 | 0.795 | -0.358; 2.756 | 0.60 |
| S | 0.868 | 0.757 | -0.616; 2.352 | 0.32 |
| Site(NNP) | 1.119 | 1.073 | -0.983; 3.222 | 0.23 |

^*^ β_0_ = model intercept; A = age; S = sex; G = generations in captivity; Site = release site, FP was the reference category
